# Supplementary material for: Trend towards virtual and hybrid conferences may be an effective climate change mitigation strategy
Source: Nat Commun. 2021 Dec 16;12:7324. doi: 10.1038/s41467-021-27251-2 (PMC8677730; doi:10.1038/s41467-021-27251-2)
Supplement: Supplementary file 1 — Supplementary Info file [file 41467_2021_27251_MOESM1_ESM.pdf]

Supplementary Information for

Trend towards virtual and hybrid conferences may be an effective climate change mitigation strategy

Authors: Yanqiu Tao[1], Debbie Steckel[2], Jiří Jaromír Klemeš[3], Fengqi You[1,4,5*]

Affiliation:

[1] Systems Engineering, Cornell University, Ithaca, New York, 14853, USA

[2] The American Center for Life Cycle Assessment, Bethesda, Maryland, 20824, USA

[3] Sustainable Process Integration Laboratory – SPIL, NETME Centre, Faculty of Mechanical Engineering, Brno University of Technology - VUT Brno, Technická 2896/2, 616 69, Brno, Czech Republic

[4] Robert Frederick Smith School of Chemical and Biomolecular Engineering, Cornell University, Ithaca, New York, 14853, USA

[5] Cornell Atkinson Center for Sustainability, 340 Tower Road Cornell University Ithaca, NY 14853, USA

Figs. S1 to S19

[*] Corresponding author. Robert Frederick Smith School of Chemical and Biomolecular Engineering, Cornell University, Ithaca, New York, 14853, USA. Phone: (607) 255-1162; Fax: (607) 255-9166; Email: fengqi.you@cornell.edu

Tables S1 to S4

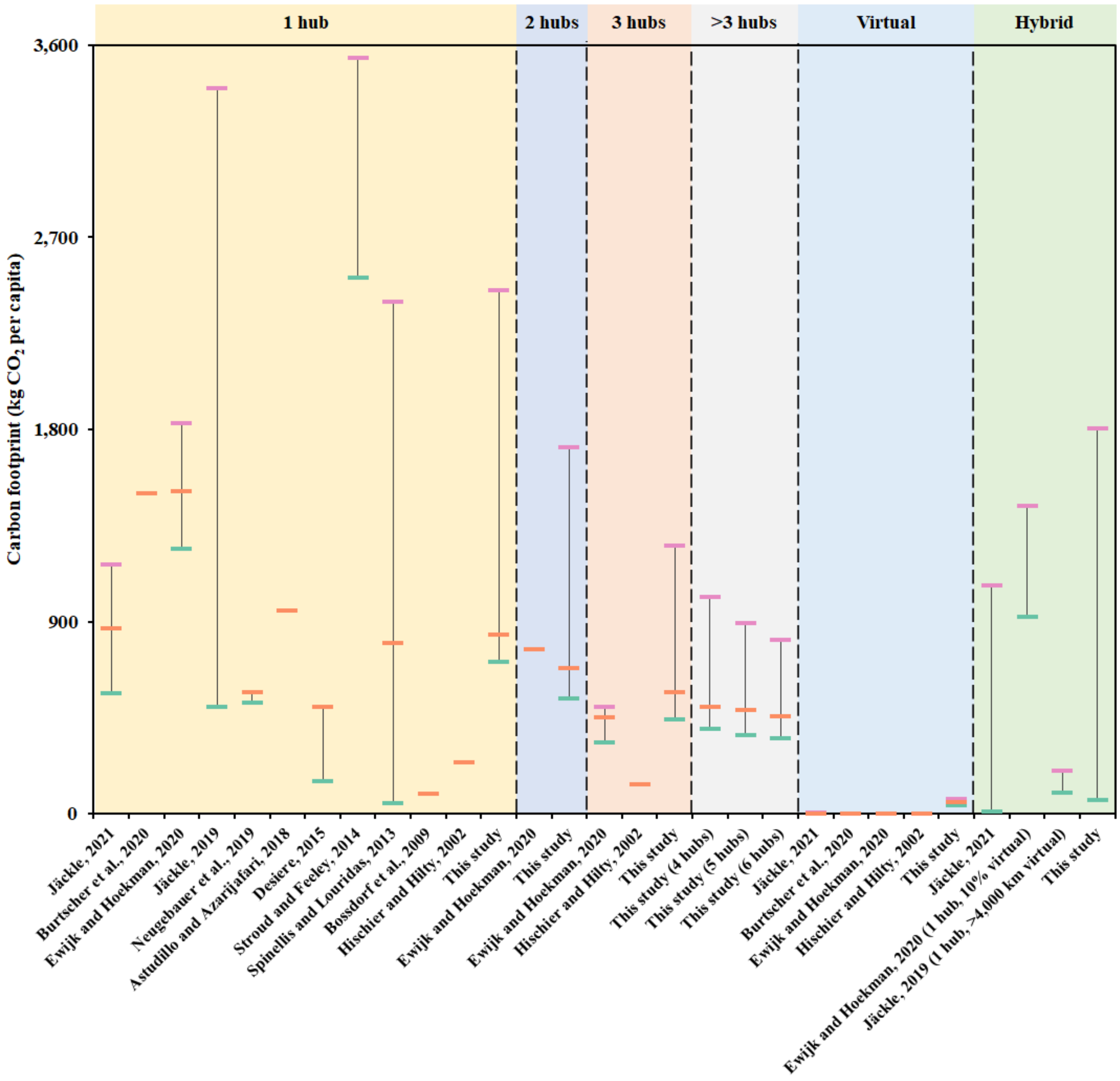

Supplementary Fig. 1 Comparison of per capita carbon footprint results from previous literature and this study. By taking the whole life cycle of a virtual conference into consideration, the carbon footprint of the virtual conference in this study is substantially higher than those in other studies[1-4]. The carbon footprint of 1-hub in-person conferences is within the range of values reported by existing studies. Only a few studies investigated the carbon footprint of multi-hub in-person conferences. The carbon footprint of 1-hub and 2-hub in-person conferences from Ewijk and Hoekman[3] is higher than that from our study because the participants of that conference are more geographically distributed than those in this study. Due to the same reason, the carbon

footprint of the 3-hub in-person conference from Ewijk and Hoekman[3] is significantly reduced and becomes lower than our result.

Supplementary Table 1 Literature comparison on the life cycle assessment (LCA) of conferences. Abbreviations: GWP, global warming potential; ICT, information and communication technology; CED, cumulative energy demand; LCIA, life cycle impact assessment; NH, New Hampshire; US, the United States; UK, the United Kingdom.

Author, Year	Conference mode	Number of conference hubs	Number of participants	Geospatial distribution of participants	Length (Day)	Location	GWP per capita (kg CO_2 eq)	Life cycle stages	LCIA method
Jäckle, 2021[1]	In-person, virtual, hybrid	1	2208	-	5	Innsbruck, Austria	In-person: 566-1166<br>Virtual: 0.35 (0.03-5.87)	Catering, accommodation, transportation	GWP
Burtscher et al., 2020[2]	In-person, virtual	0,1	1240 (in-person)<br>1777 (virtual)	84% from Europe (in-person)	5	Lyon, France<br>Virtual	In-person: 1500<br>Virtual: 0.33	Transportation<br>ICT	GWP
Ewijk and Hoekman, 2020[3]	In-person, virtual, hybrid (1 hub, 10% virtual)	0,1,2,3	625 (in-person)<br>588 (in-person)<br>401 (in-person)	Dependent on conference site†	-	Chicago, US<br>Surrey, UK<br>Ulsan, South Korea<br>Virtual	In-person (1 hub): 1513 (1240-1830)<br>In-person (2 hubs): 770<br>In-person (3 hubs): 450 (330-500)<br>Hybrid: 920-1440<br>0 (virtual)	Transportation	GWP
Jäckle, 2019[5]	In-person, hybrid	1	1930	Europe: 89%<br>North America: 4%<br>Asia: 4%<br>Rest of World: 3%	3-4	Bordeaux, France<br>Glasgow, UK<br>Montreal, Canada<br>Prague, Czechia<br>Oslo, Norway<br>Hamburg, Germany	500-3400	Transportation	GWP
Neugebauer et al., 2019[6]	In-person	1	800	Europe: 85%<br>Asia: 5%<br>North America: 4%<br>South America: 3%<br>Rest of World: 3%	3	Aachen, Germany	518-570	Preparation, execution, catering, accommodation, transportation	CML2001, USEtox
Astudillo and Azarijafari, 2018[7]	In-person	1	228	North America: 87%<br>Europe: 5%<br>Asia: 4%<br>Rest of World: 4%	3	Portsmouth, NH, US	952	Transportation, catering, accommodation	GWP
Desiere, 2015[8]	In-person	1	646	Europe: 85%	-	Ljubljana, Slovenia	150-498	Transportation	GWP

† Most participants are from the continent where the conference is held

				US: 4%<br>Japan: 2%<br>Rest of World: 9%		Stuttgart, Germany			
Stroud and Feeley, 2014[9]	In-person	1	311<br>207<br>385<br>406<br>450	-	-	Canary Island, Spain<br>Merida, Mexico<br>Crete, Greece<br>Miami, FL, US<br>Bayreuth, Germany	2580-3220<br>3000-3540<br>2510-3020<br>2560-2910<br>2540-2720	Transportation	GWP
Spinellis and Louridas, 2013[10‡]	In-person	-	-	-	-	Multi-location multi-year	801 (48 – 2396)	Transportation	GWP
Bossdorf et al., 2009[11]	In-person	1	125	-	3	Bern, Switzerland	92	Preparation, execution, catering, accommodation, transportation	GWP
Hischier and Hilty, 2002[4]	In-person, virtual	0,1,3	308 (in-person)<br>1000 (virtual)	-	3	Zurich, Switzerland	In-person: 240<br>Virtual: 2.5<br>Hybrid: 137	Organization, materials, and transportation	Eco-Indicator 99<br>UBP 97<br>CO_2
This study	In-person, virtual, hybrid	0,1,2,3,4,5,6	536	North America: 88%<br>Europe: 8%<br>Rest of World: 4%		Optimal location	55 (virtual)<br>455-840 (in-person)<br>64-1804 (hybrid)	Preparation, execution, catering, accommodation, transportation ICT	GWP<br>CED<br>ReCiPe

‡ This paper randomly sampled proceeding conference papers from Scopus

Supplementary Table 2 The geographic distribution of participants for the 2020 American Center for Life Cycle Assessment virtual conference.

Participant's origin	Percentage
United States	76.76%
Belgium	0.52%
Brazil	1.04%
Canada	11.49%
Switzerland	1.31%
Costa Rica	0.26%
Germany	1.31%
Ecuador	0.26%
Egypt	0.26%
Spain	0.78%
United Kingdom	2.35%
India	0.52%
Iran	0.26%
Italy	1.04%
Japan	0.52%
Kuwait	0.26%
Netherlands	0.52%
France	0.26%
Sweden	0.26%

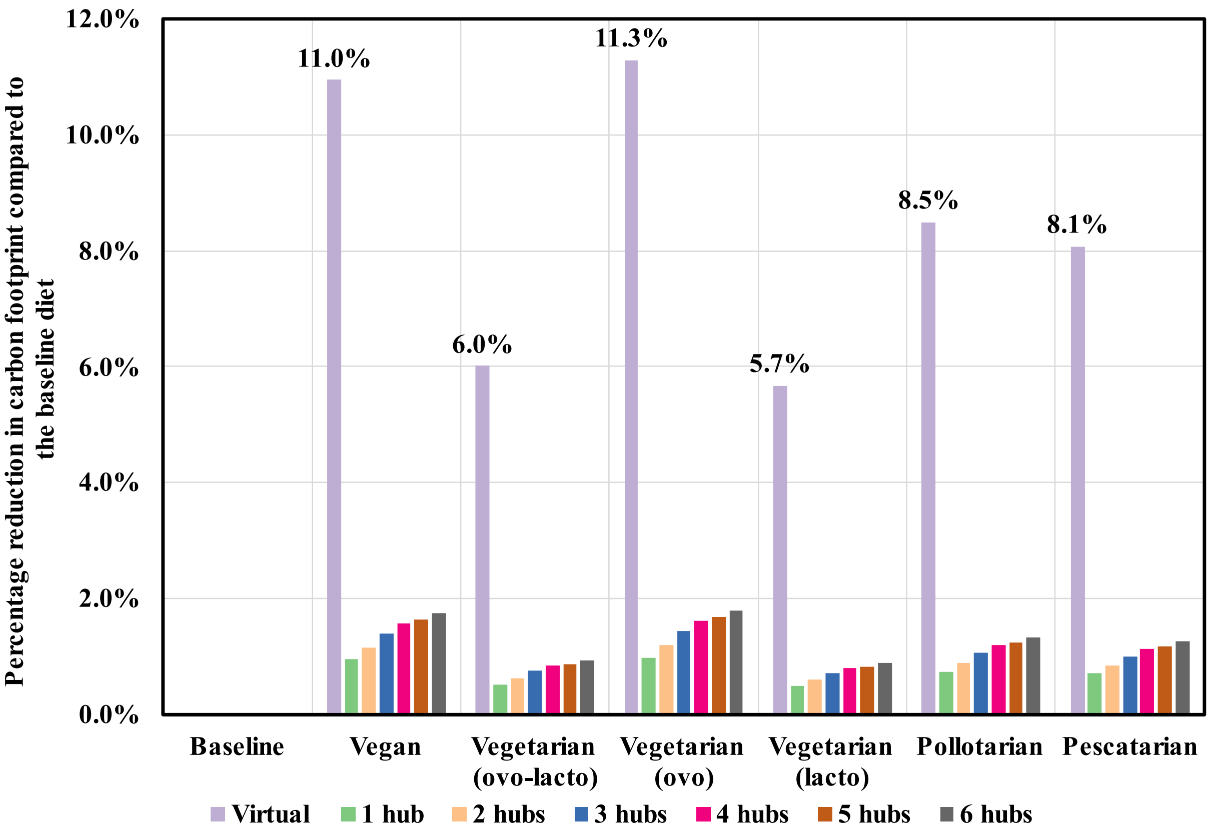

Supplementary Fig. 2 Sensitivity analysis of dietary types. The baseline diet is set as the average food supply per capita for different countries[12,13]. Both the amount and composition of the food supply are summarized in Supplementary Table S3. For the sensitivity analysis, we consider a vegan diet as replacing all animal-related food products with soybean products products; an ovo-lacto vegetarian diet that replaces the livestock meat, fish, animal fats with soybean products; an ovo vegetarian diet that replaces the livestock meat, fish, animal fats, and dairy with soybean products; a lacto vegetarian diet that replaces the livestock meat, fish, animal fats, and eggs with soybean products; a pollotarian diet that replaces all the livestock meat with poultry meat; a pescatarian diet that replaces all the livestock meat with fish. The ovo vegetarian diet performs better in mitigating carbon footprint than other types of diets.

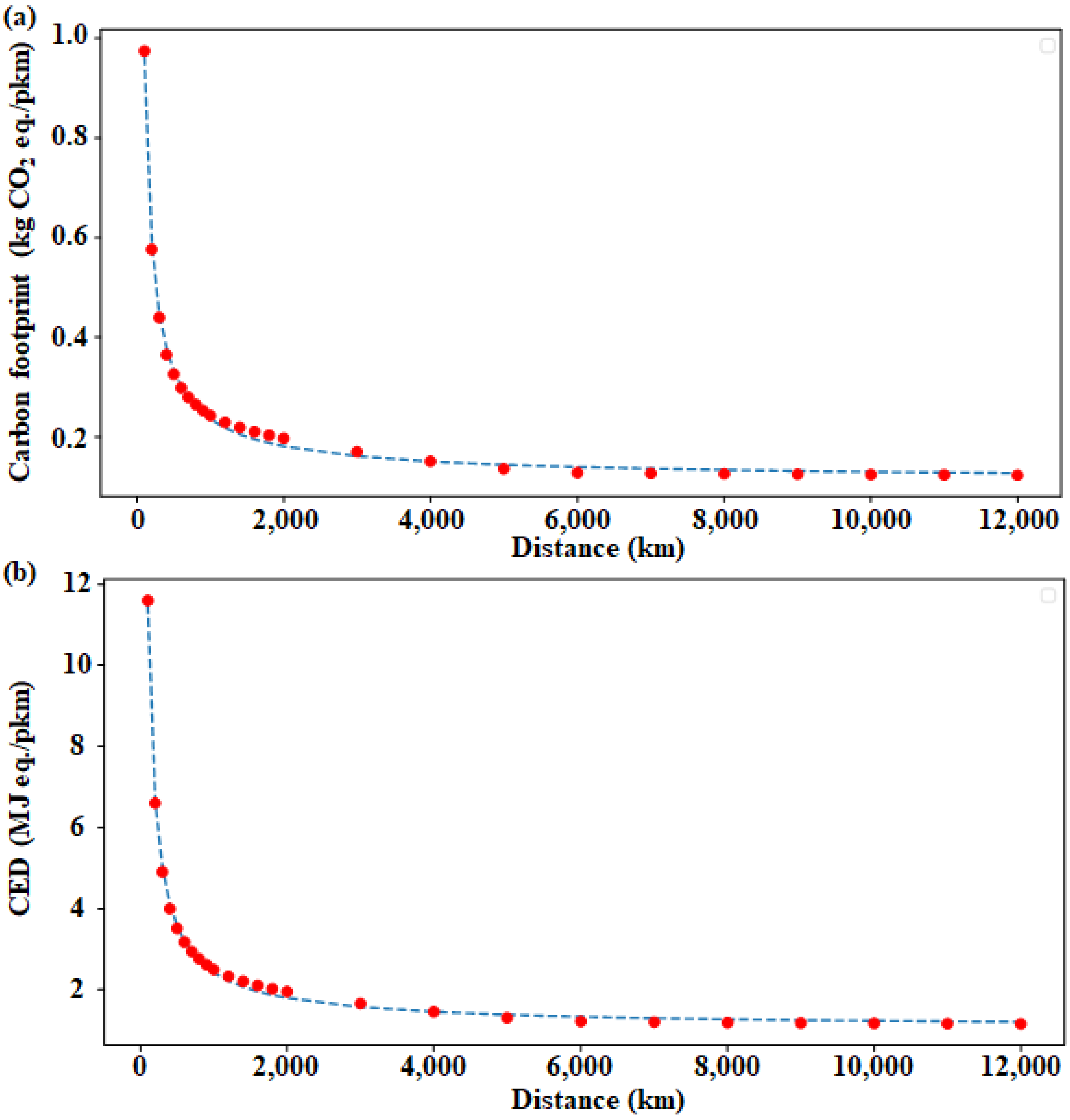

Supplementary Fig. 3 The carbon footprint and cumulative energy demand (CED) per passenger-km (pkm) of flight. We fit the characterization factors of air transportation over distance from Cox et al.[14] to polynomial functions for the year 2020. The values of power, slope, intercept, and R^2 are summarized in Supplementary Table S3.

Supplementary Table 3 Parameters of the fitted power functions for the characterization factors of air transportation over distance from Cox et al.[14].

Impact category	Power	Slope	Intercept	R^2
Global Warming Potential (kg CO_2 eq per passenger km)	-8.39E-01	4.06E+01	1.12E-01	9.97E-01
Cumulative Energy Demand, Non-Renewable (MJ per passenger km)	-8.82E-01	6.08E+02	1.05E+00	9.98E-01
Agricultural Land Occupation Potential (m^2yr per passenger km)	-8.94E-01	4.29E-01	2.52E-04	9.99E-01
Fossil Depletion Potential (kg oil eq per passenger km)	-8.80E-01	1.39E+01	2.48E-02	9.98E-01
Freshwater Ecotoxicity Potential (kg 14-DCB eq per passenger km)	-9.19E-01	1.08E-01	8.57E-05	9.99E-01
Freshwater Eutrophication Potential (kg P eq per passenger km)	-9.57E-01	3.43E-03	1.30E-06	1.00E+00
Human Toxicity Potential (kg 1,4 DB eq per passenger km)	-9.83E-01	5.92E+00	2.07E-03	1.00E+00
Ionising Radiation Potential (kg U235 eq per passenger km)	-9.02E-01	3.67E+00	4.79E-03	9.99E-01
Marine Ecotoxicity Potential (kg 14-DCB eq per passenger km)	-9.30E-01	1.06E-01	6.63E-05	9.99E-01
Marine Eutrophication Potential (kg N eq per passenger km)	-9.20E-01	6.03E-02	1.38E-04	1.00E+00
Metal Depletion Potential (kg Fe eq per passenger km)	-9.15E-01	3.58E-01	3.45E-04	9.99E-01
Natural Land Transformation Potential (m^2 per passenger km)	-8.81E-01	1.48E-02	2.60E-05	9.98E-01
Ozone Depletion Potential (kg CFC11 eq per passenger km)	-8.78E-01	7.00E-06	1.26E-08	9.98E-01
Particulate Matter Formation Potential (kg PM10 eq per passenger km)	-9.36E-01	4.31E-02	2.75E-05	1.00E+00
Photochemical Oxidant Formation Potential (kg NMVOC per passenger km)	-9.58E-01	1.65E-01	6.36E-05	1.00E+00
Terrestrial Acidification Potential (kg SO_2 eq per passenger km)	-9.57E-01	2.32E-03	1.15E-06	1.00E+00
Terrestrial Ecotoxicity Potential (kg 14-DCB eq per passenger km)	-9.57E-01	2.32E-03	1.15E-06	1.00E+00
Urban Land Occupation Potential (m^2yr per passenger km)	-9.66E-01	3.78E-01	1.30E-04	1.00E+00
Water Depletion Potential (m^3 H_2O per passenger km)	-9.43E-01	1.17E-01	7.29E-05	1.00E+00

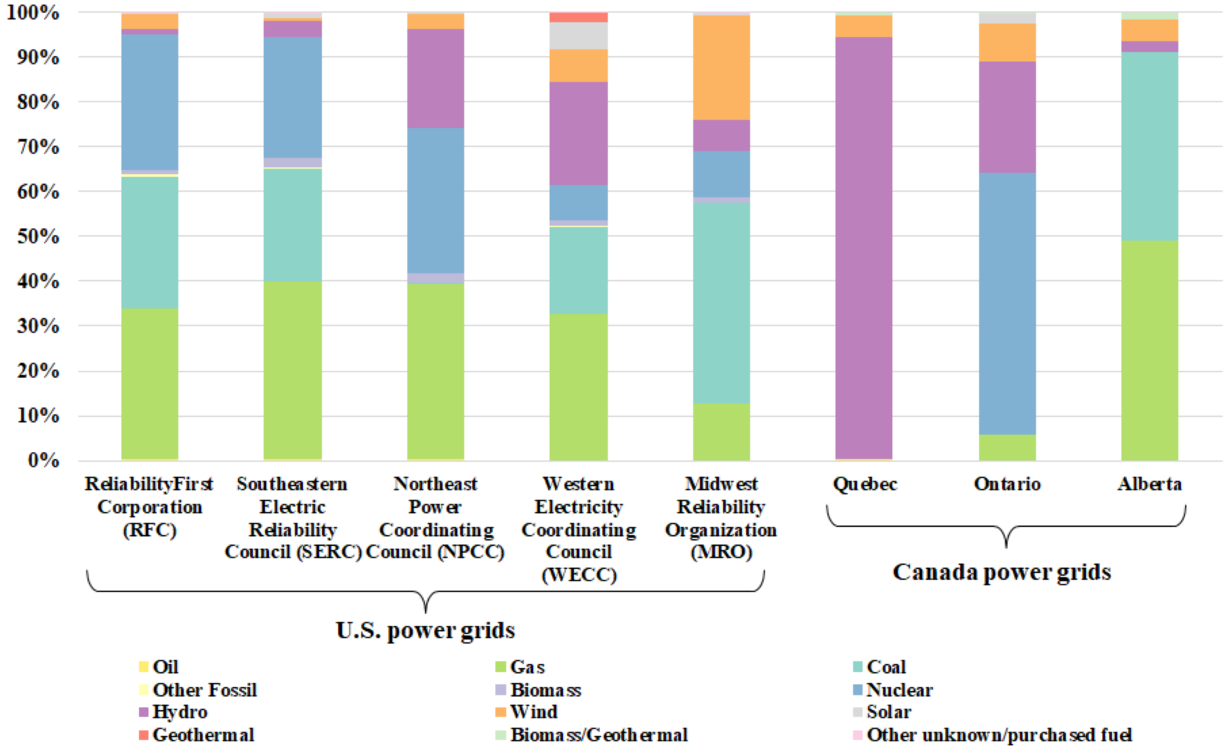

Supplementary Fig. 4 Energy sources of power grids in the United States (U.S.) and Canada subregions where 84% of participants are from[15,16].

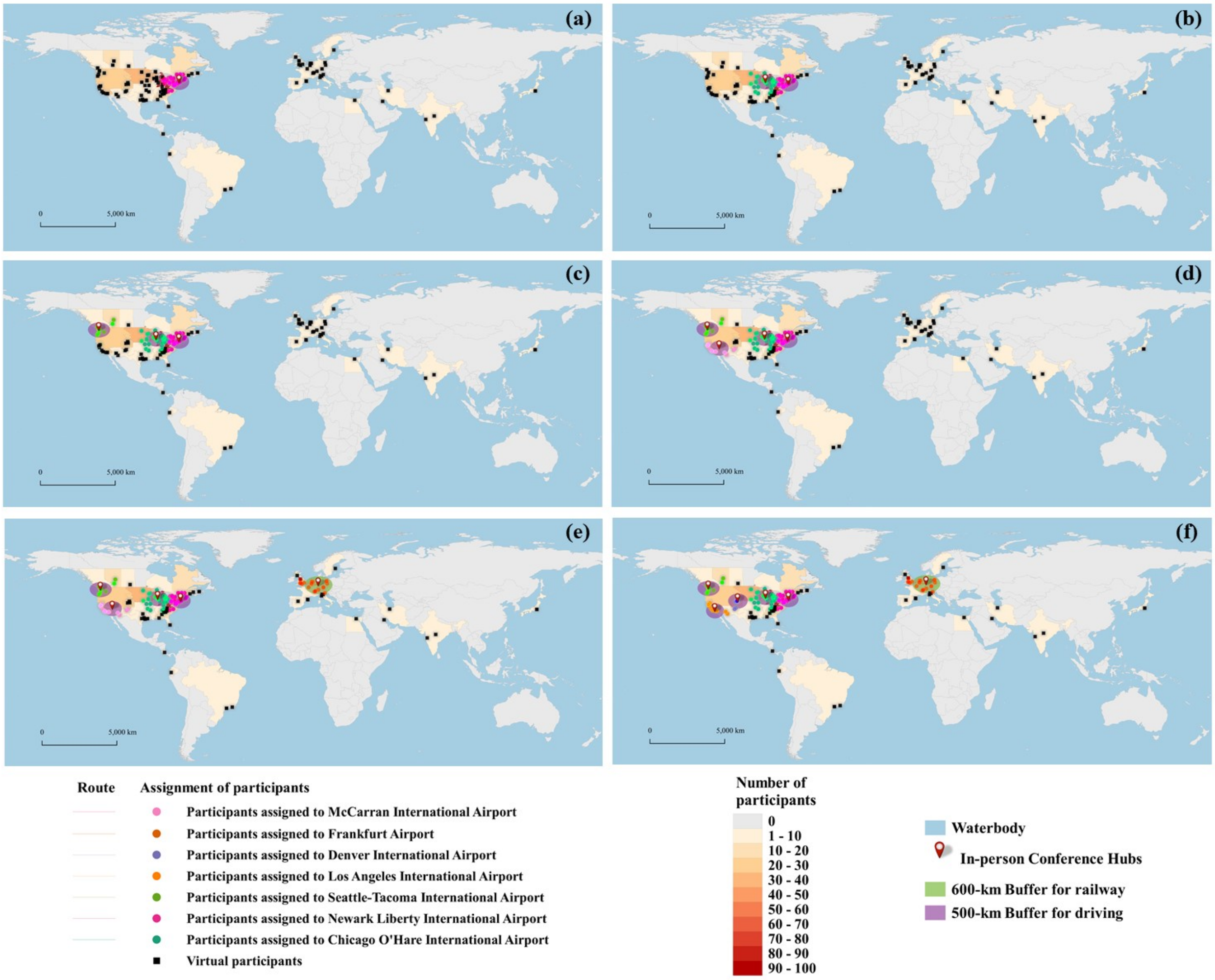

Supplementary Fig. 5 Map of transportation routes and hub decisions for hybrid scenarios with maximum travel distance of 1,000 km. a, 1-hub hybrid conference. **b**, 2-hub hybrid conference. **c**, 3-hub hybrid conference. **d**, 4-hub hybrid conference. **e**, 5-hub hybrid conference. **f**, 6-hub hybrid conference. Dots represent the origins of participants, and lines represent the route from their origins to their assigned conference hubs. Their colors indicate the assignments of participants to the conference hubs. Color scale indicates the number of participants in each region around the world. These regions are divided according to the regional datasets for electricity production in Ecoinvent[17].

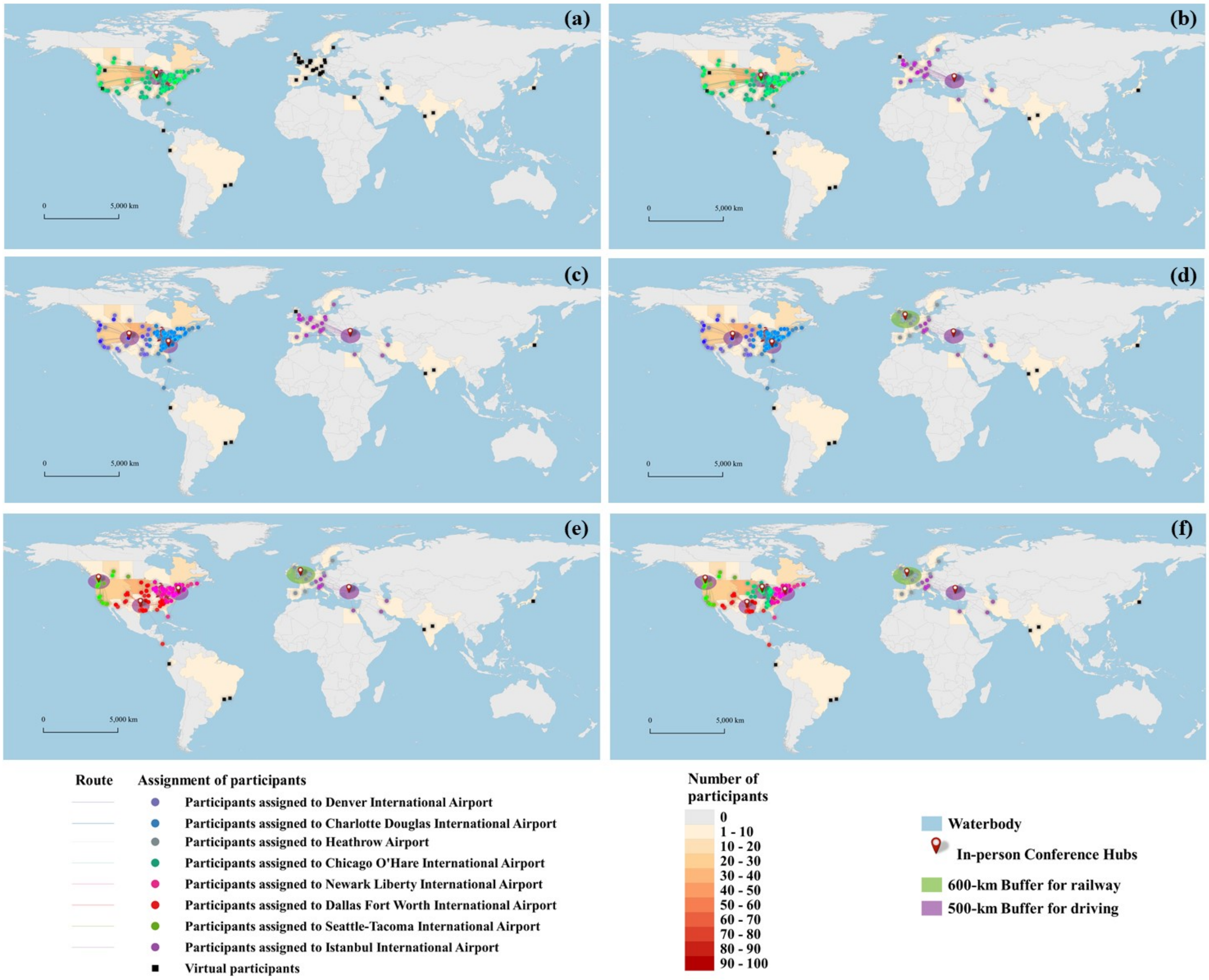

Supplementary Fig. 6 Map of transportation routes and hub decisions for hybrid scenarios with maximum travel distance of 3,000 km. a, 1-hub hybrid conference. **b**, 2-hub hybrid conference. **c**, 3-hub hybrid conference. **d**, 4-hub hybrid conference. **e**, 5-hub hybrid conference. **f**, 6-hub hybrid conference. Dots represent the origins of participants, and lines represent the route from their origins to their assigned conference hubs. Their colors indicate the assignments of participants to the conference hubs. Color scale indicates the number of participants in each region around the world. These regions are divided according to the regional datasets for electricity production in Ecoinvent[17].

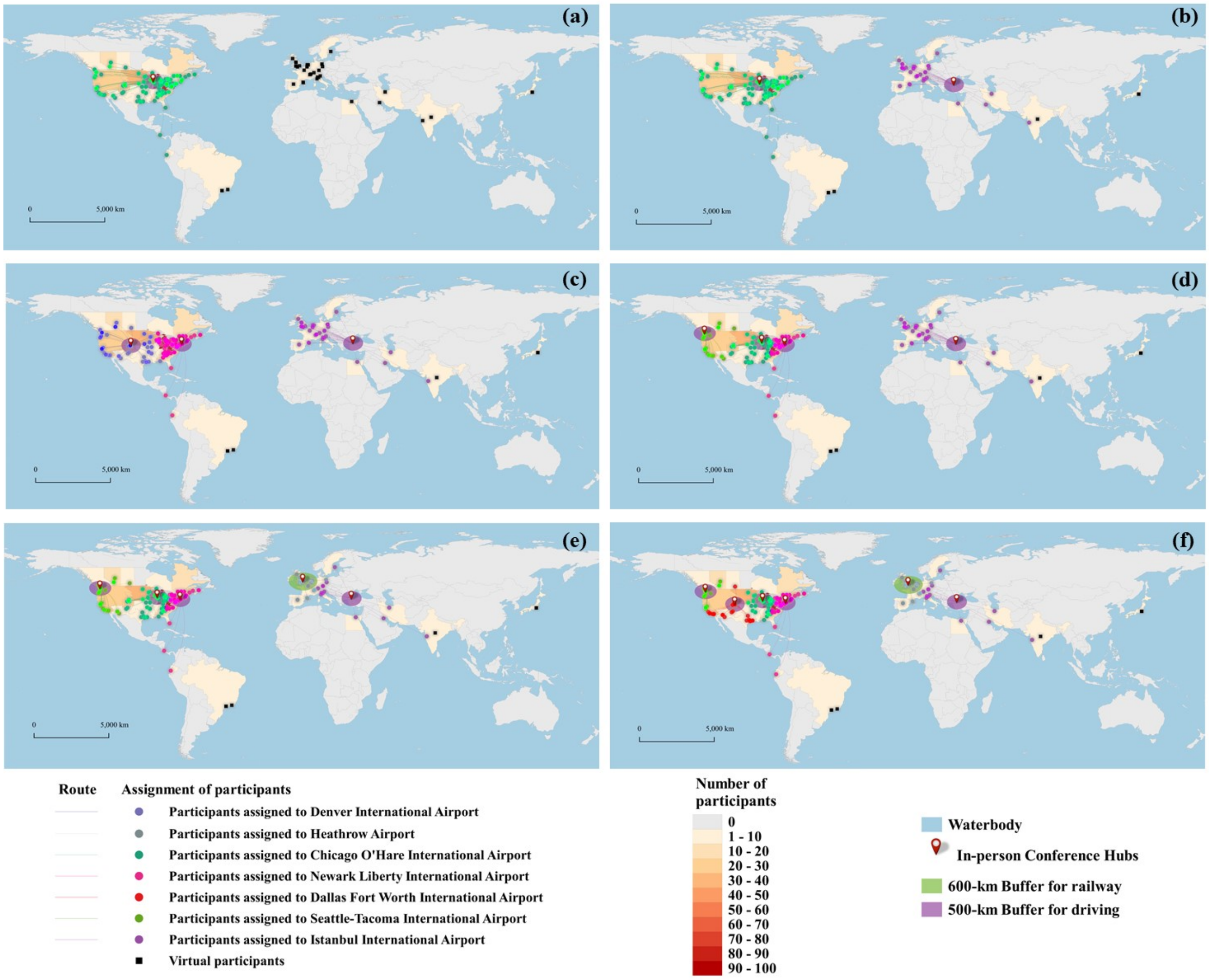

Supplementary Fig. 7 Map of transportation routes and hub decisions for hybrid scenarios with maximum travel distance of 5,000 km. **a**, 1-hub hybrid conference. **b**, 2-hub hybrid conference. **c**, 3-hub hybrid conference. **d**, 4-hub hybrid conference. **e**, 5-hub hybrid conference. **f**, 6-hub hybrid conference. Dots represent the origins of participants, and lines represent the route from their origins to their assigned conference hubs. Their colors indicate the assignments of participants to the conference hubs. Color scale indicates the number of participants in each region around the world. These regions are divided according to the regional datasets for electricity production in Ecoinvent[17].

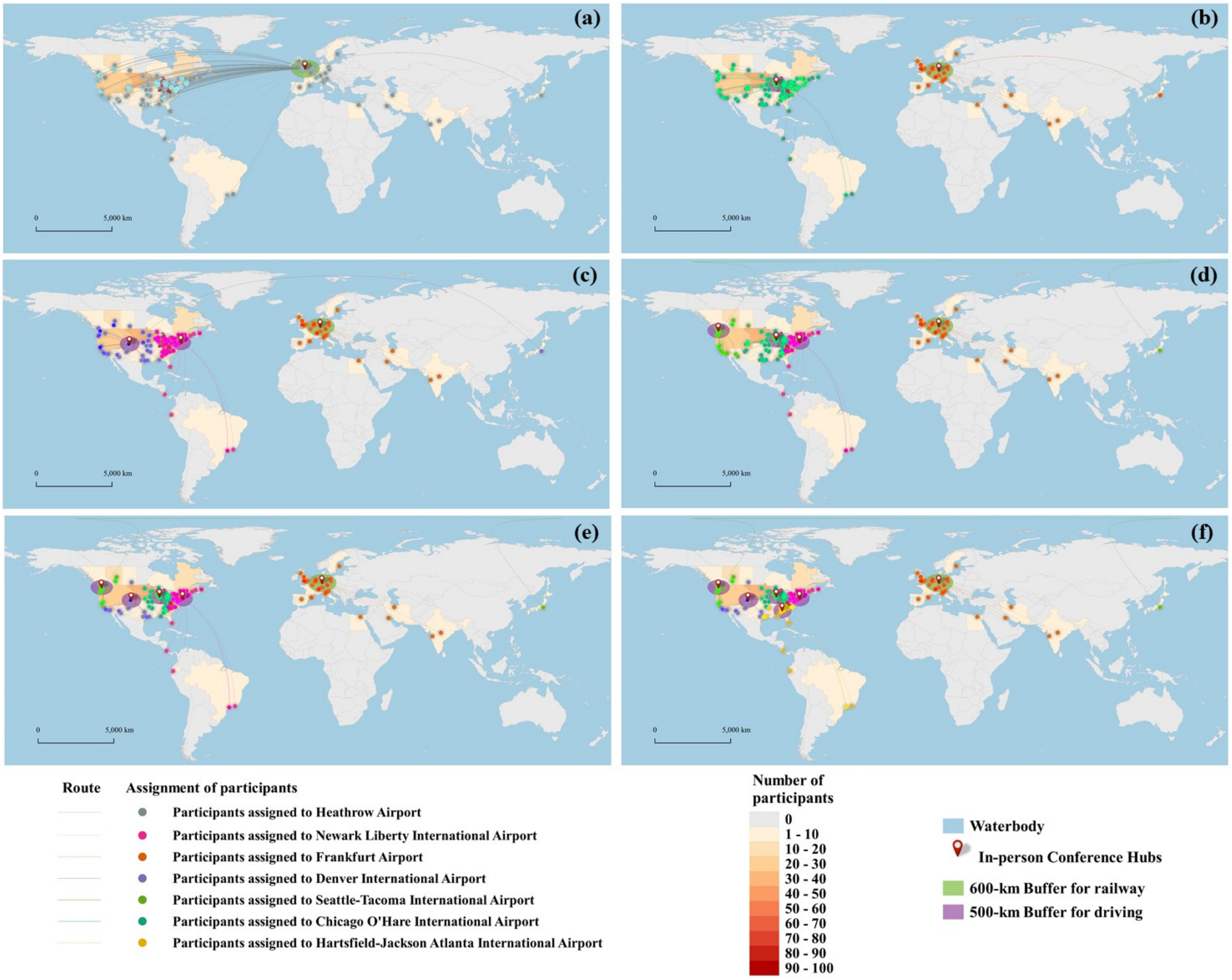

Supplementary Fig. 8 Map of transportation routes and hub decisions for hybrid scenarios with maximum travel distance of 10,000 km. a, 1-hub hybrid conference. **b**, 2-hub hybrid conference. **c**, 3-hub hybrid conference. **d**, 4-hub hybrid conference. **e**, 5-hub hybrid conference. **f**, 6-hub hybrid conference. Dots represent the origins of participants, and lines represent the route from their origins to their assigned conference hubs. Their colors indicate the assignments of participants to the conference hubs. Color scale indicates the number of participants in each region around the world. These regions are divided according to the regional datasets for electricity production in Ecoinvent[17].

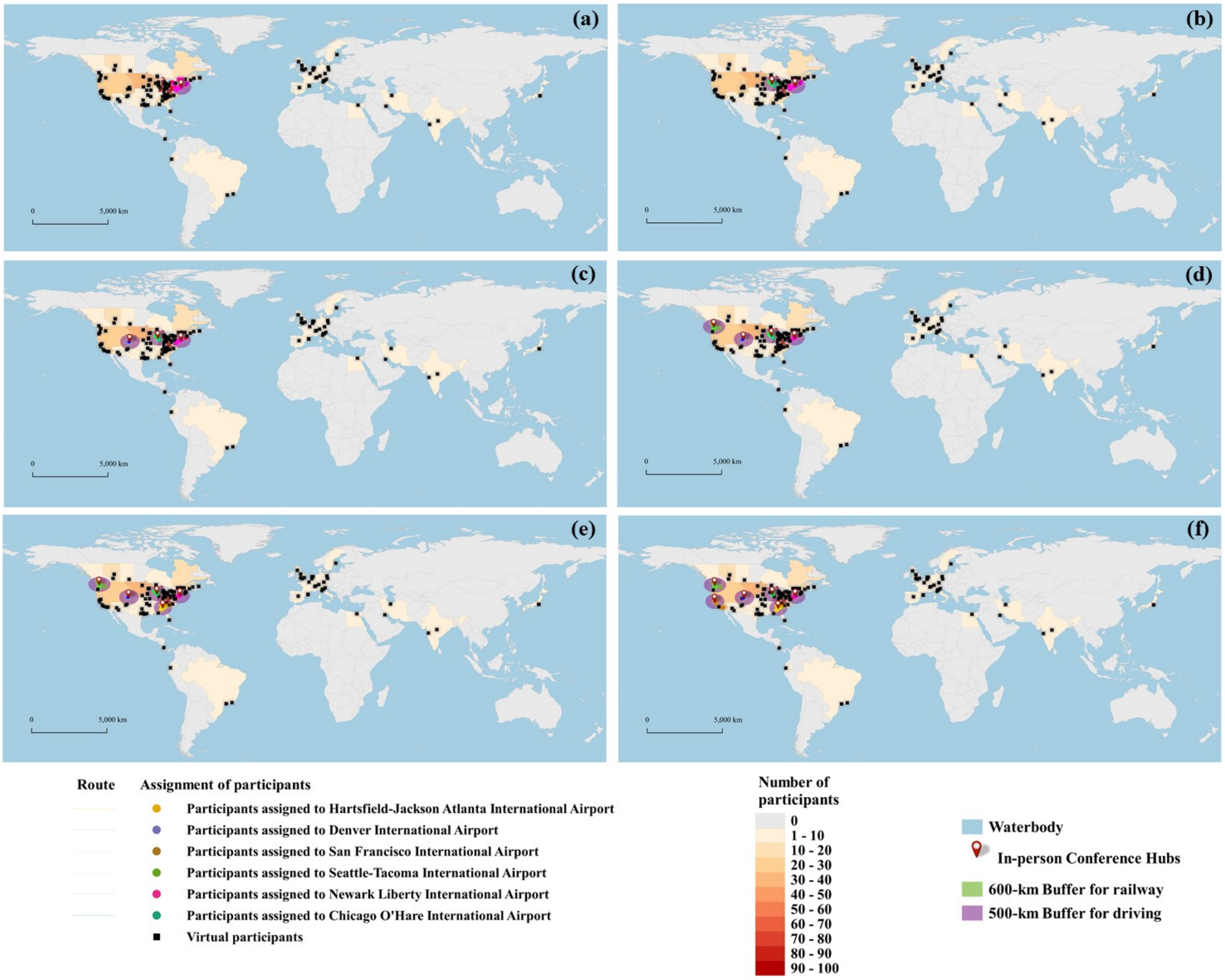

Supplementary Fig. 9 Map of transportation routes and hub decisions for hybrid scenarios with 70% maximum virtual participation. **a**, 1-hub hybrid conference. **b**, 2-hub hybrid conference. **c**, 3-hub hybrid conference. **d**, 4-hub hybrid conference. **e**, 5-hub hybrid conference. **f**, 6-hub hybrid conference. Dots represent the origins of participants, and lines represent the route from their origins to their assigned conference hubs. Their colors indicate the assignments of participants to the conference hubs. Color scale indicates the number of participants in each region around the world. These regions are divided according to the regional datasets for electricity production in Ecoinvent[17].

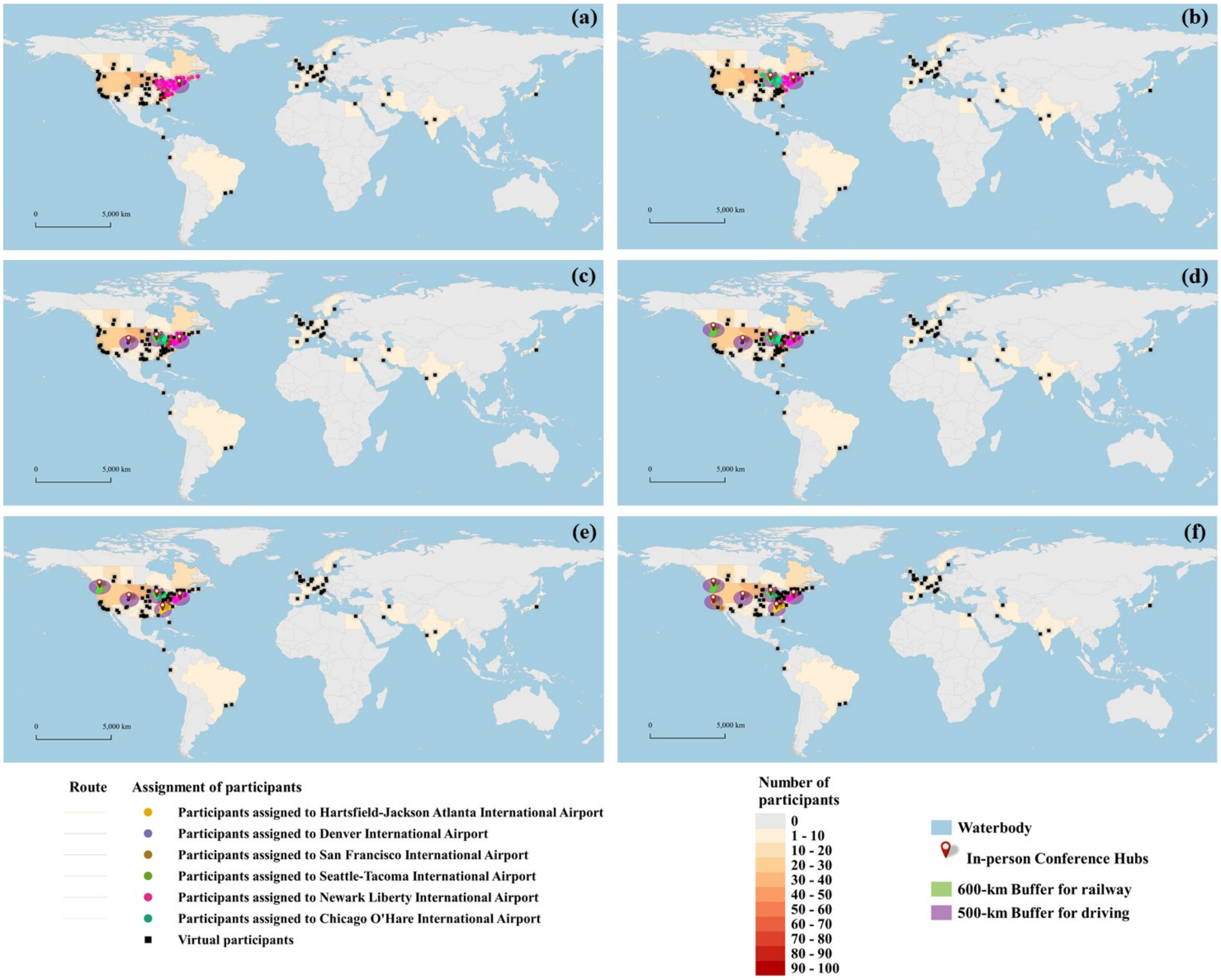

Supplementary Fig. 10 Map of transportation routes and hub decisions for hybrid scenarios with 50% maximum virtual participation. **a**, 1-hub hybrid conference. **b**, 2-hub hybrid conference. **c**, 3-hub hybrid conference. **d**, 4-hub hybrid conference. **e**, 5-hub hybrid conference. **f**, 6-hub hybrid conference. Dots represent the origins of participants, and lines represent the route from their origins to their assigned conference hubs. Their colors indicate the assignments of participants to the conference hubs. Color scale indicates the number of participants in each region around the world. These regions are divided according to the regional datasets for electricity production in Ecoinvent[17].

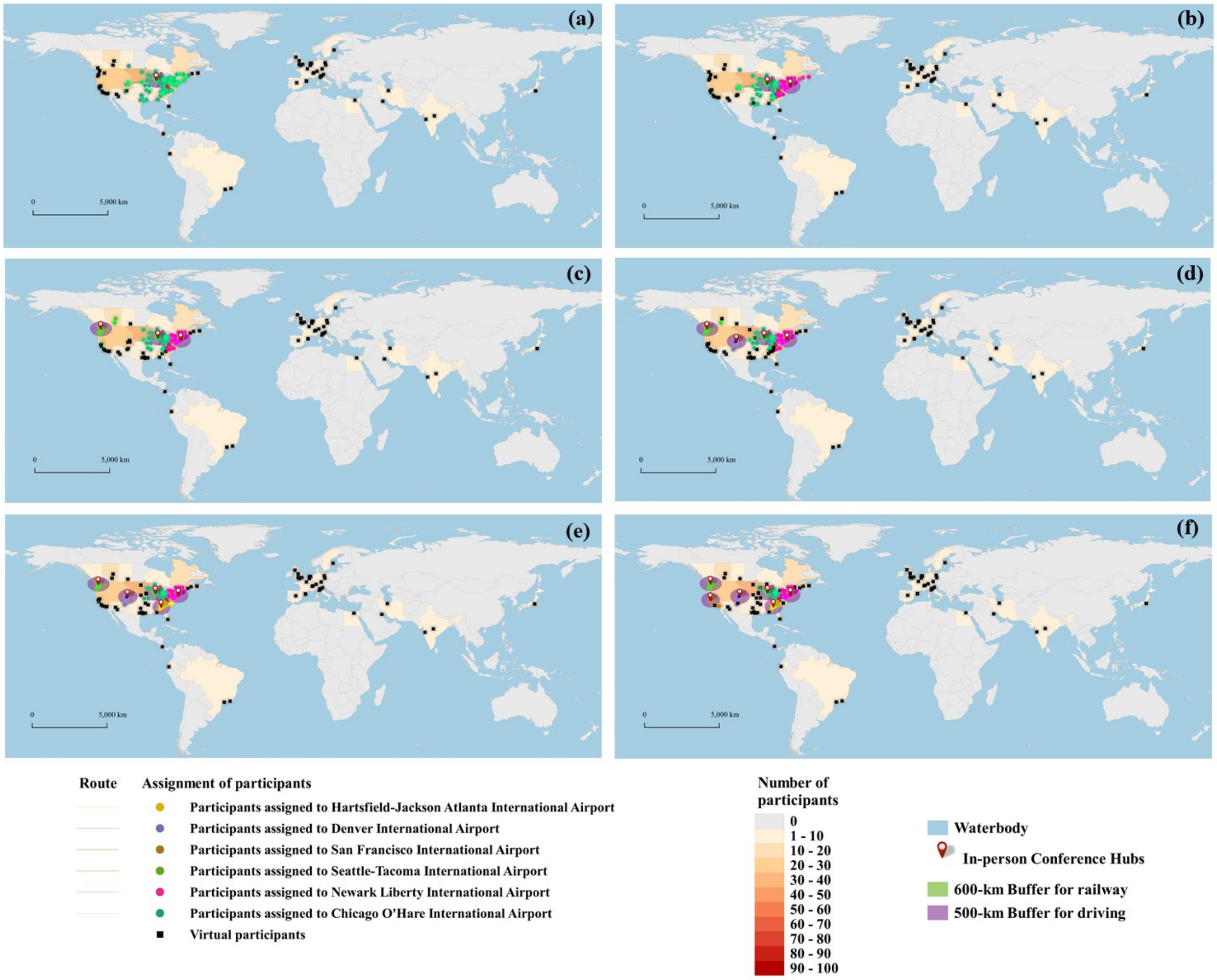

Supplementary Fig. 11 Map of transportation routes and hub decisions for hybrid scenarios with 30% maximum virtual participation. **a**, 1-hub hybrid conference. **b**, 2-hub hybrid conference. **c**, 3-hub hybrid conference. **d**, 4-hub hybrid conference. **e**, 5-hub hybrid conference. **f**, 6-hub hybrid conference. Dots represent the origins of participants, and lines represent the route from their origins to their assigned conference hubs. Their colors indicate the assignments of participants to the conference hubs. Color scale indicates the number of participants in each region around the world. These regions are divided according to the regional datasets for electricity production in Ecoinvent[17].

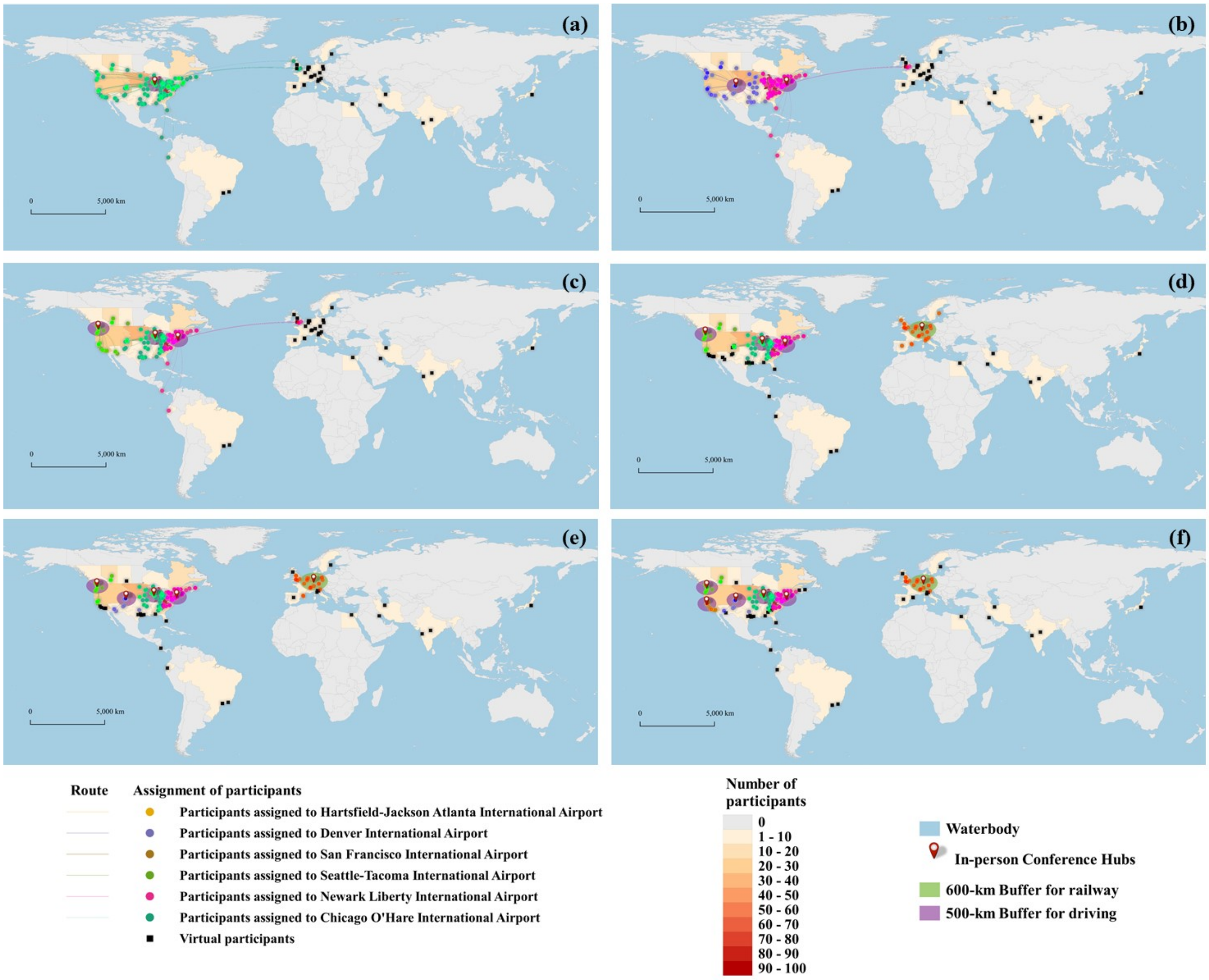

Supplementary Fig. 12 Map of transportation routes and hub decisions for hybrid scenarios with 10% maximum virtual participation. **a**, 1-hub hybrid conference. **b**, 2-hub hybrid conference. **c**, 3-hub hybrid conference. **d**, 4-hub hybrid conference. **e**, 5-hub hybrid conference. **f**, 6-hub hybrid conference. Dots represent the origins of participants, and lines represent the route from their origins to their assigned conference hubs. Their colors indicate the assignments of participants to the conference hubs. Color scale indicates the number of participants in each region around the world. These regions are divided according to the regional datasets for electricity production in Ecoinvent[17].

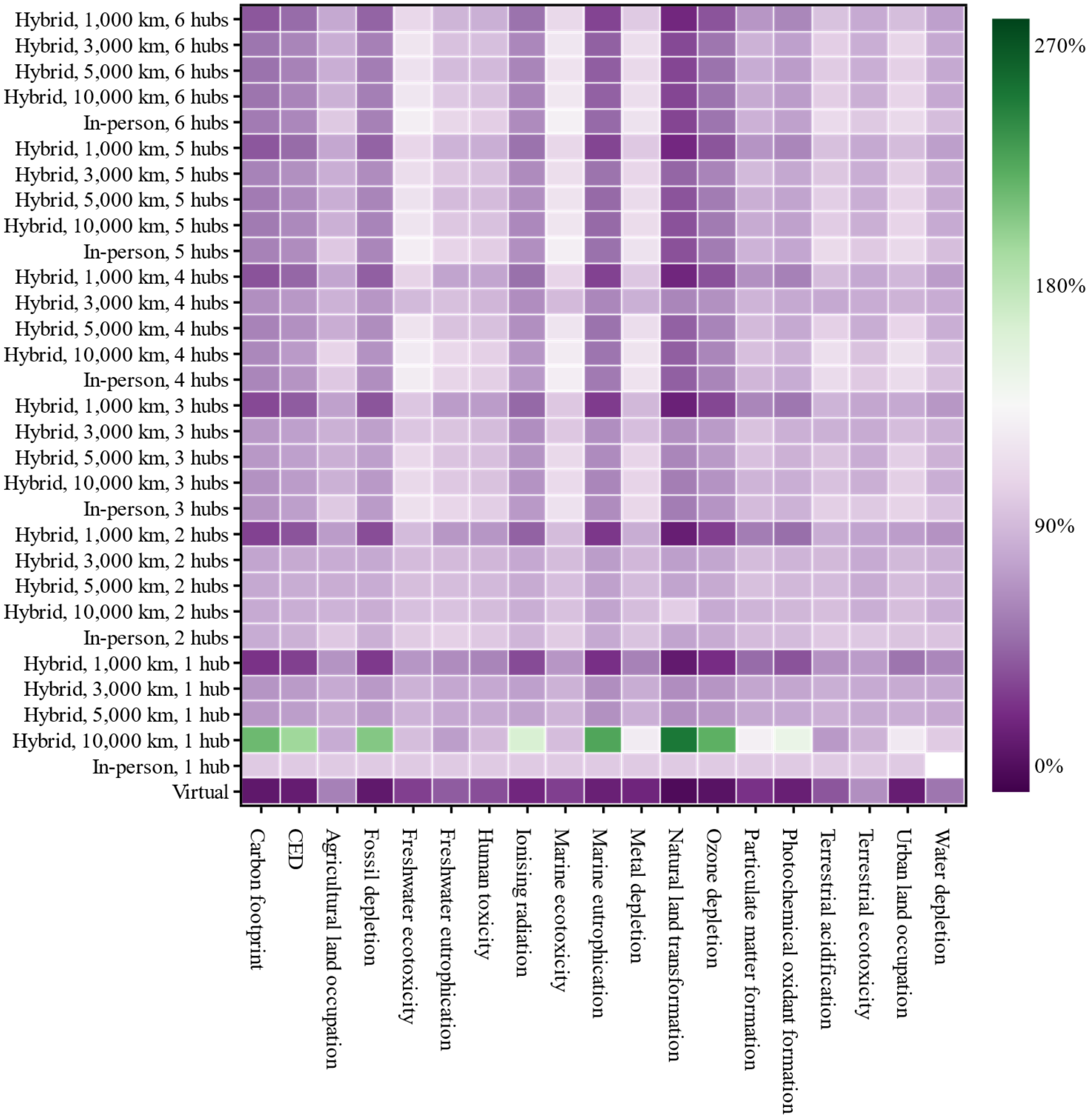

Supplementary Fig. 13 Carbon footprint, cumulative energy demand (CED), and 17 ReCiPe midpoint indicators of the virtual, in-person, and "maximum travel distance" scenarios with 1 to 6 hubs for an average participant. The y axis lists the mode, specific constraint (i.e., maximum travel distance), and the number of conference hubs for each scenario. From top to bottom, scenarios with the same amount of conference hubs are ranked by their virtual participation level in descending order. The percentages are computed based on the environmental impacts of the 1-hub in-person conference scenario.

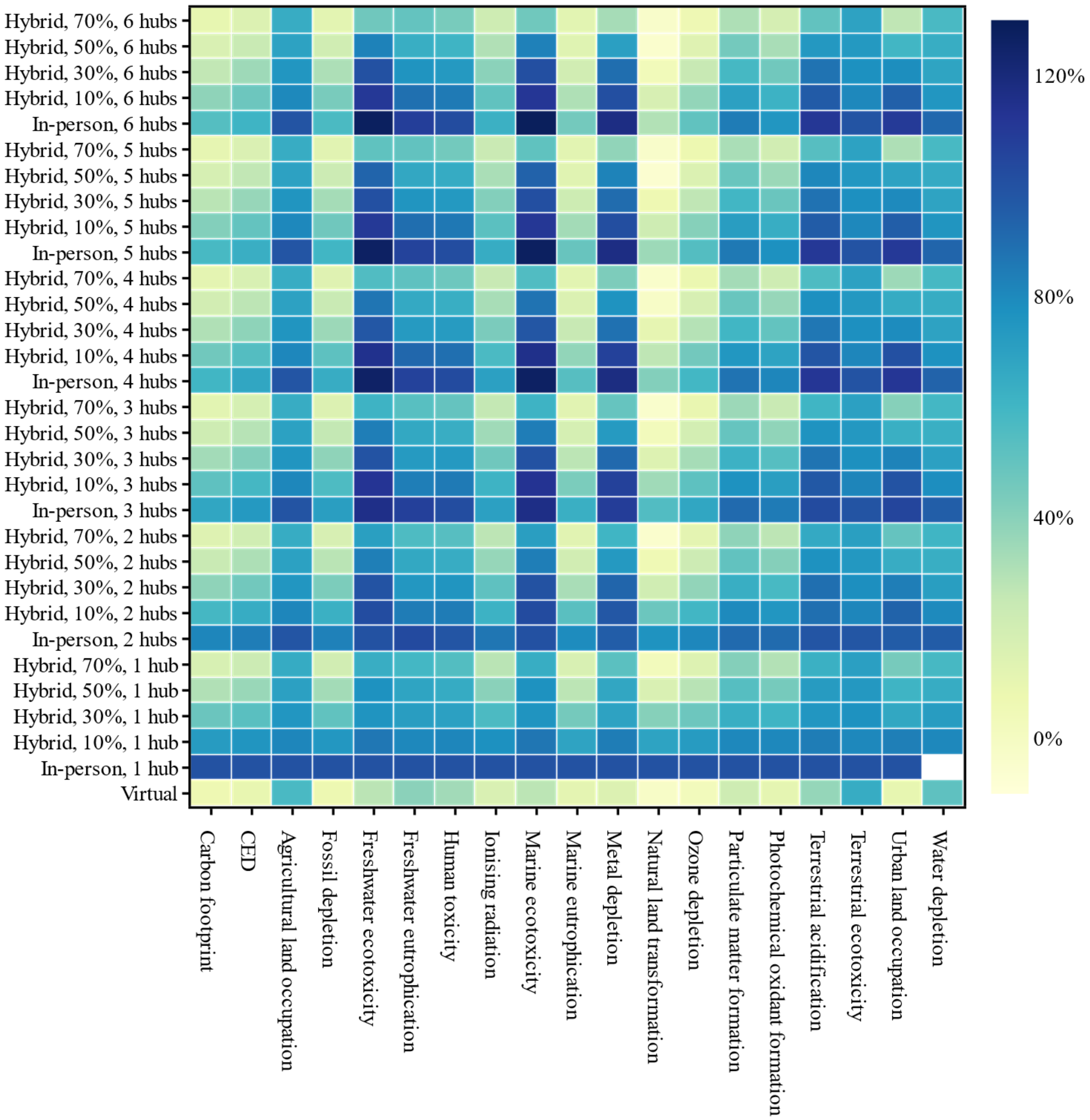

Supplementary Fig. 14 Carbon footprint, cumulative energy demand (CED), and 17 ReCiPe midpoint indicators of the virtual, in-person, and "maximum virtual participation" scenarios with 1 to 6 hubs for an average participant. The y axis lists the mode, specific constraint (i.e., maximum virtual participation), and the number of conference hubs for each scenario. From top to bottom, scenarios with the same amount of conference hubs are ranked by their virtual participation level in descending order. The percentages are computed based on the environmental impacts of the 1-hub in-person conference scenario.

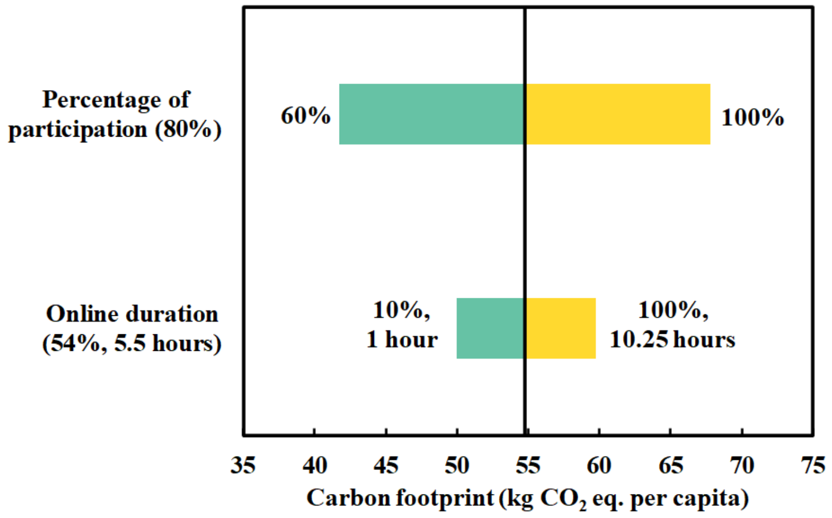

Supplementary Fig. 15 Sensitivity analysis on percentage of participation and online duration for virtual conferences.

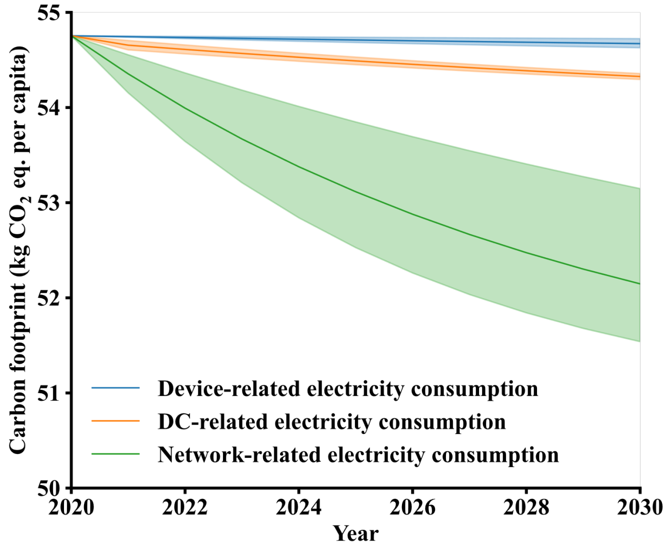

Supplementary Fig. 16 Sensitivity analysis on the device-related, data centre (DC)-related, and network-related electricity consumption based on the annual energy efficiency improvement rate of the worst- or best-case scenario[18]. Three lines represent the baseline estimation on energy efficiency improvement; upper bound of each band represents the worst-case scenario of energy efficiency improvement; and lower bound of each band represents the best-case scenario of energy efficiency improvement.

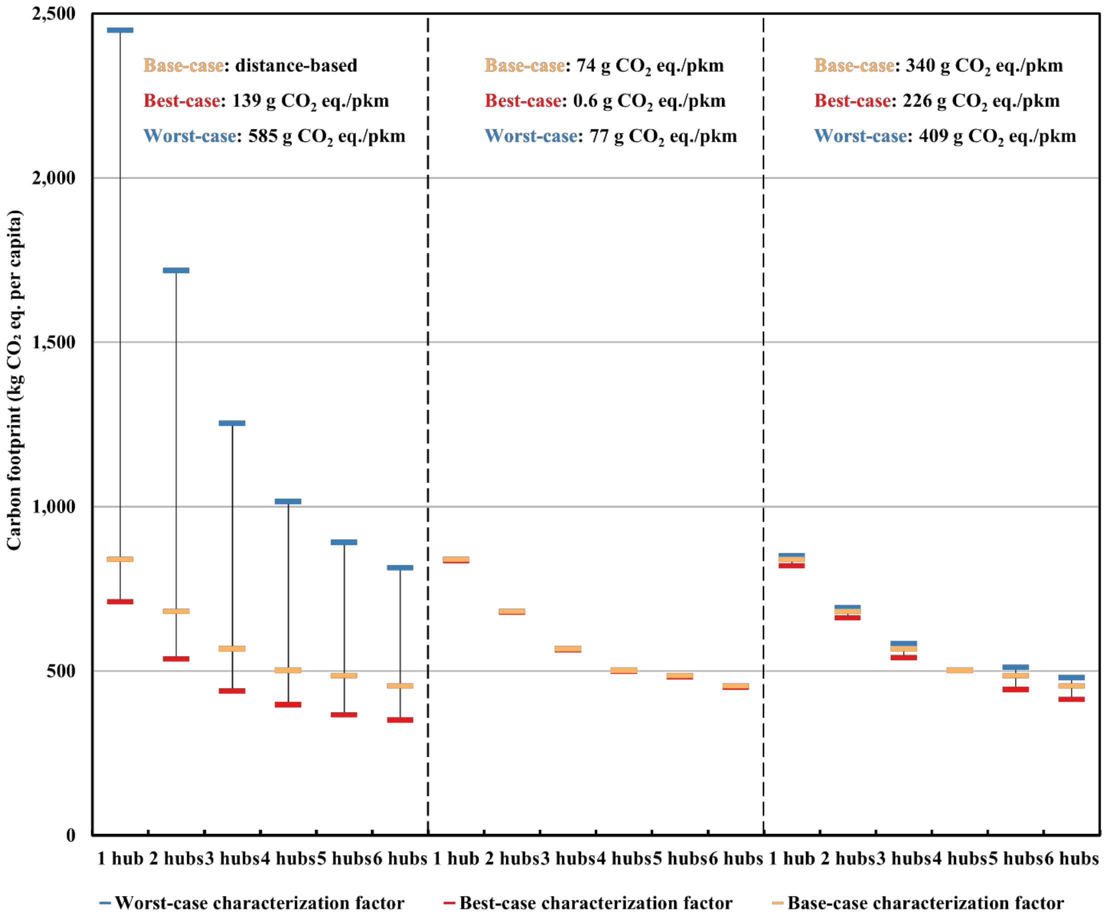

Supplementary Fig. 17 Sensitivity analysis on characterization factors of three transportation modes. In-person scenarios with 1 to 6 hubs for an average participant are assessed. The base-case characterization factor for the carbon footprint of air transportation is obtained from Cox et al.[14]; the best-case and worst-case characterization factors are obtained from the UK Department for Business, Energy & Industrial Strategy (International flights for an average economy-class passenger and long-haul flights for an average first-class passenger, respectively)[19]. The base-, best- and worst-case characterization factors for the carbon footprint of rail transportation and driving are from the Ecoinvent database (Supplementary Table S4) and the Network for Transport Measures[17,20]. Specifically, the base-, best- and worst-case characterization factors for rail transportation are chosen among the country-specific processes for passenger train or high-speed passenger train, while the selection is conducted among passenger cars with different size and fuel for driving.

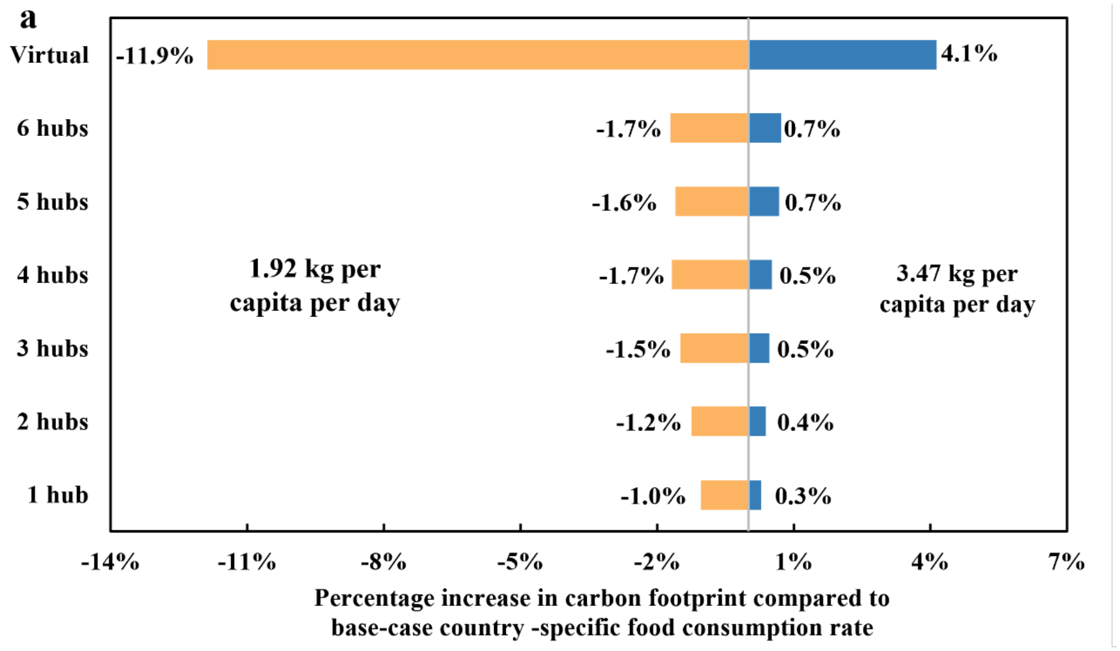

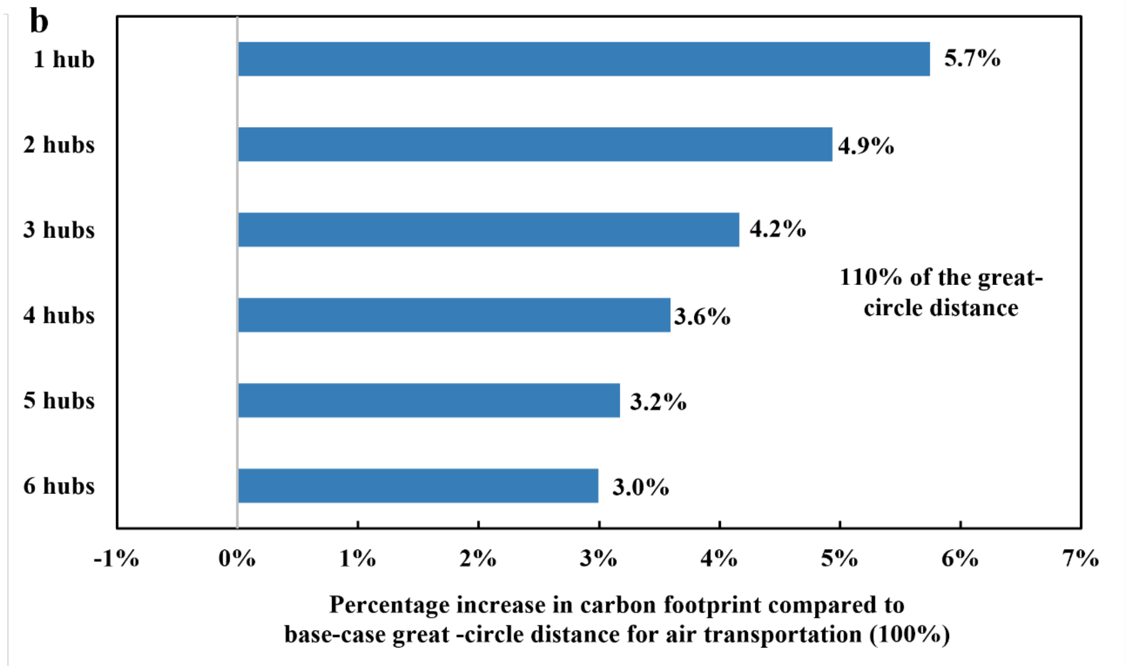

Supplementary Fig. 18 Sensitivity analysis on food consumption rate and air transportation distance. Virtual and in-person scenarios with 1 to 6 hubs for an average participant are assessed. **a,** Food consumption rate. The base-case food consumption rate is obtained from FAOSTAT[12,13], as shown in Table 4. The maximum and minimum total food consumption rate in Table 4 is considered as the best case and worst case. **b,** Air transportation distance. Great-circle distance is considered as the base case, and 110% of the great-circle distance is considered as the worst case[21].

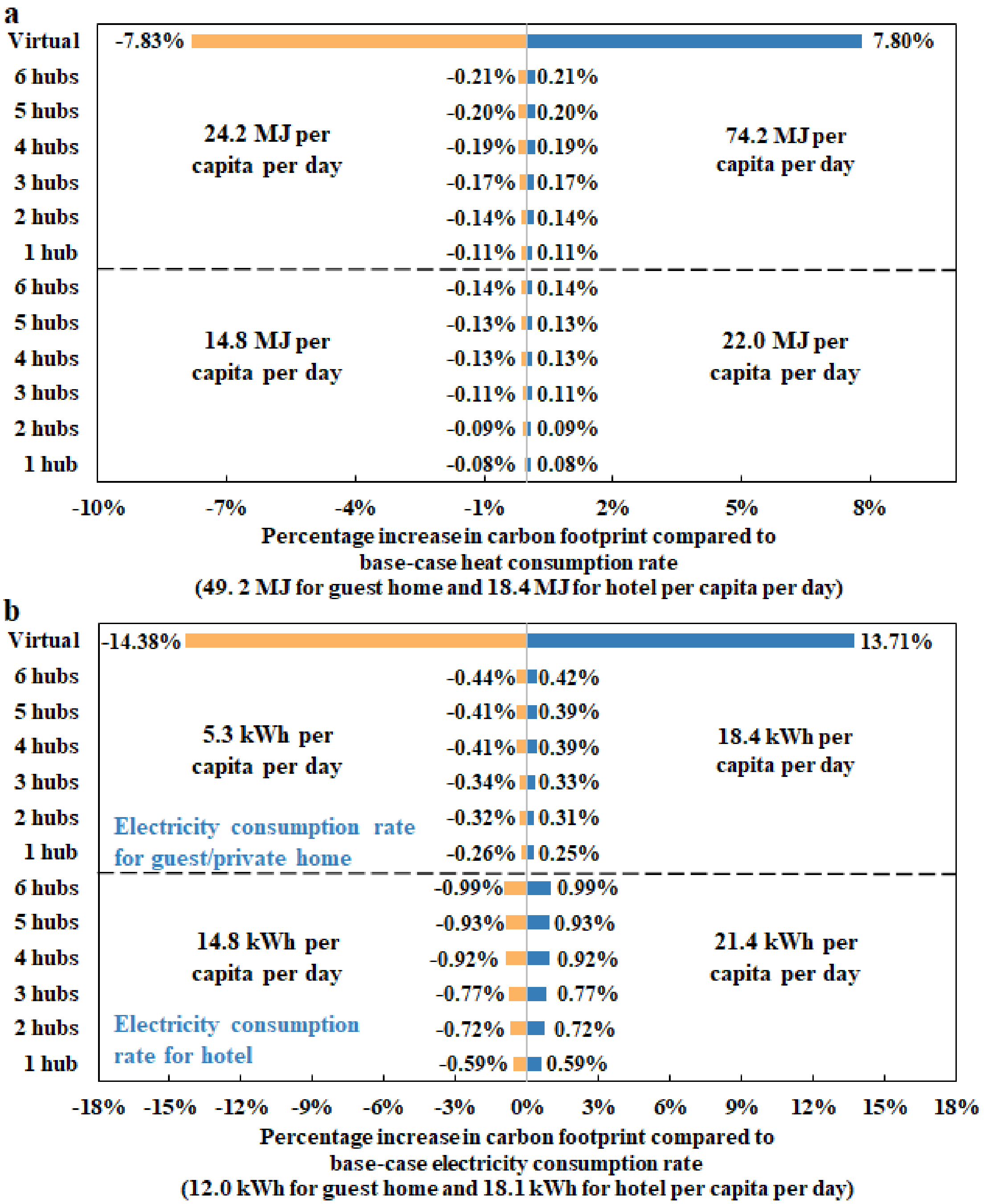

Supplementary Fig. 19 Sensitivity analysis on heat and electricity consumption rate at guest/private home and hotel. Virtual and in-person scenarios with 1 to 6 hubs for an average participant are assessed. **a,** Heat consumption rate. **b,** Electricity consumption rate. The base-, worst-, best-case value of heat and electricity consumption rate at the hotel is obtained from Filimonau et al.[22]; The base-, worst-, best-case value of heat and electricity consumption rate at the hotel is obtained from the U.S. Energy Information Administration[23,24].

Supplementary Table 4 Comparison of characterization factors for the carbon footprint of air transportation, rail transport, and driving. The base-case characterization factor for the carbon footprint of air transportation is obtained from Cox et al.[14]; the best-case and worst-case characterization factors are obtained from the UK Department for Business, Energy & Industrial Strategy (International flights for an average economy-class passenger and long-haul flights for an average first-class passenger, respectively)[19]. The base-, best- and worst-case characterization factors for the carbon footprint of rail transportation and driving are from the Ecoinvent database and the Network for Transport Measures[17,20]. Specifically, the base-, best- and worst-case characterization factors for rail transportation are chosen among the country-specific processes for passenger train or high-speed passenger train, while the selection is conducted among passenger cars with different sizes and fuel for driving.

Category	Item	Location	Value	Unit	Case
Air transportation	Distance-based[14], see Supplementary Table S3	Swiss	-	-	Base
	International flights, economy class[19]	UK	1.39E-01	kg CO_2 eq./pkm	Best
	Long-haul flights, first class[19]	UK	5.85E-01	kg CO_2 eq./pkm	Worst
Rail transport	transport, passenger train, DE[17]	Germany	7.45E-02	kg CO_2 eq./pkm	Base
	transport, passenger train, BE[17]	Belgium	4.53E-02	kg CO_2 eq./pkm	
	transport, passenger train, FR[17]	France	2.37E-02	kg CO_2 eq./pkm	
	transport, passenger train, IT[17]	Italy	4.89E-02	kg CO_2 eq./pkm	
	transport, passenger train, RoW[17]	Rest-of-World	7.66E-02	kg CO_2 eq./pkm	Worst
	transport, passenger train, high-speed, DE[17]	Germany	6.10E-02	kg CO_2 eq./pkm	
	transport, passenger train, high-speed, FR[17]	France	2.00E-02	kg CO_2 eq./pkm	
	transport, passenger train, high-speed, IT[17]	Italy	4.88E-02	kg CO_2 eq./pkm	
	transport, passenger train, high-speed, RoW[17]	Rest-of-World	7.14E-02	kg CO_2 eq./pkm	
	High spend train with green electricity[20]	-	5.70E-04	kg CO_2 eq./pkm	Best
	High speed train[20]	EU	3.76E-02	kg CO_2 eq./pkm	
	Inter city train with green electricity[20]	-	7.10E-04	kg CO_2 eq./pkm	
	Inter city train[20]	EU	4.63E-02	kg CO_2 eq./pkm	
	Regional train with green electricity[20]	-	8.00E-04	kg CO_2 eq./pkm	
	Regional train[20]	EU	5.25E-02	kg CO_2 eq./pkm	
Driving	market for transport, passenger car, small size, petrol, EURO 5, GLO[17]	Global	2.71E-01	kg CO_2 eq./pkm	
	market for transport, passenger car, small size, diesel, EURO 5, GLO[17]	Global	2.34E-01	kg CO_2 eq./pkm	
	market for transport, passenger car, medium size, petrol, EURO 5, GLO[17]	Global	3.40E-01	kg CO_2 eq./pkm	Base
	market for transport, passenger car, medium size, diesel, EURO 5, GLO[17]	Global	3.09E-01	kg CO_2 eq./pkm	
	market for transport, passenger car, large size, petrol, EURO 5, GLO[17]	Global	4.09E-01	kg CO_2 eq./pkm	Worst
	market for transport, passenger car, large size, diesel, EURO 5, GLO[17]	Global	3.86E-01	kg CO_2 eq./pkm	
	market for transport, passenger car, electric, GLO[17]	Global	2.26E-01	kg CO_2 eq./pkm	Best

References

1 Jäckle, S. Reducing the Carbon Footprint of Academic Conferences by Online Participation: The Case of the 2020 Virtual European Consortium for Political Research General Conference. *PS: Political Science & Politics* **54**, 456-461, (2021).

2 Burtscher, L. et al. The carbon footprint of large astronomy meetings. *Nature Astronomy* **4**, 823-825, (2020).

3 van Ewijk, S. & Hoekman, P. Emission reduction potentials for academic conference travel *Journal of Industrial Ecology* **n/a**, (2020).

4 Hischier, R. & Hilty, L. Environmental impacts of an international conference. *Environmental Impact Assessment Review - ENVIRON IMPACT ASSESS REV* **22**, 543-557, (2002).

5 Jäckle, S. WE have to change! The carbon footprint of ECPR general conferences and ways to reduce it. *European Political Science* **18**, 630-650, (2019).

6 Neugebauer, S., Bolz, M., Mankaa, R. & Traverso, M. How sustainable are sustainability conferences? – comprehensive Life Cycle Assessment of an International Conference Series in Europe. *Journal of Cleaner Production* **242**, 118516, (2019).

7 Astudillo, M. F. & AzariJafari, H. Estimating the global warming emissions of the LCAXVII conference: connecting flights matter. *The International Journal of Life Cycle Assessment* **23**, 1512-1516, (2018).

8 Desiere, S. The Carbon Footprint of Academic Conferences: Evidence from the 14th EAAE Congress in Slovenia. *EuroChoices* **15**, 56-61, (2016).

9 Stroud, J. T. & Feeley, K. J. Responsible academia: optimizing conference locations to minimize greenhouse gas emissions. *Ecography* **38**, 402-404, (2015).

10 Spinellis, D. & Louridas, P. The Carbon Footprint of Conference Papers. *PLOS ONE* **8**, e66508, (2013).

11 Bossdorf, O., Parepa, M. & Fischer, M. Climate-neutral ecology conferences: just do it! *Trends in ecology & evolution* **25**, 61, (2009).

12 FAOSTAT. Food Supply - Livestock and Fish Primary Equivalent, (2013) (Accessed 04/19/2021) http://www.fao.org/faostat/en/#data/CL.

13 FAOSTAT. Food Supply - Crops Primary Equivalent, (2013) (Accessed 04/19/2021) http://www.fao.org/faostat/en/#data/CC.

14 Cox, B., Jemiolo, W. & Mutel, C. Life cycle assessment of air transportation and the Swiss commercial air transport fleet. *Transportation Research Part D: Transport and Environment* **58**, 1-13, (2018).

15 United States Environmental Protection Agency. Emissions & Generation Resource Integrated Database (Accessed 01/22/2021) https://www.epa.gov/egrid.

16 Canada Energy Regulator. Canada's Energy Future Data Appendices, (2020) (Accessed 07/08/2021).

17 Wernet, G. et al. The ecoinvent database version 3 (part I): overview and methodology. *Int. J. Life Cycle Assess.* **21**, 1218-1230, (2016).

18 Andrae, A. S. G. & Edler, T. On Global Electricity Usage of Communication Technology: Trends to 2030. *Challenges* **6**, (2015).

19 UK Department for Business, Energy & Industrial Strategy. Greenhouse Gas Reporting: Conversion Factors 2020, (2020) (Accessed 09/11/2021) https://www.gov.uk/government/publications/greenhouse-gas-reporting-conversion-factors-2020.

20 Network for Transport Measures. Default and Benchmark Transport Data., (2018) (Accessed 09/11/2021) https://www.transportmeasures.org/en/wiki/evaluation-transport-suppliers/train-travel-baselines-2018/.

21 Kettunen, T. et al. in *6th USA/Europe Air Traffic Management Seminar, Baltimore.* 27-30.

22 Filimonau, V., Dickinson, J., Robbins, D. & Huijbregts, M. A. J. Reviewing the carbon footprint analysis of hotels: Life Cycle Energy Analysis (LCEA) as a holistic method for carbon impact appraisal of tourist accommodation. *Journal of Cleaner Production* **19**, 1917-1930, (2011).

23 U.S. Energy Information Administration. State Energy Data System (SEDS): 1960-2019 (complete), (2021) (Accessed 09/11/2021) https://www.eia.gov/state/seds/seds-data-complete.php?sid=US.

24 U.S. Energy Information Administration. 2015 Residential Energy Consumption Survey (RECS) Data (Accessed 09/11/2021) https://www.eia.gov/consumption/residential/data/2015/.
