# Supplementary material for: Trend towards virtual and hybrid conferences may be an effective climate change mitigation strategy
Source: Nat Commun. 2021 Dec 16;12:7324. doi: 10.1038/s41467-021-27251-2 (PMC8677730; doi:10.1038/s41467-021-27251-2)
Supplement: Supplementary file 3 — Reporting Summary [file 41467_2021_27251_MOESM3_ESM.pdf]

nature portfolio

Corresponding author(s): Fengqi You

Last updated by author(s): Oct 20, 2021

Reporting Summary

Nature Portfolio wishes to improve the reproducibility of the work that we publish. This form provides structure for consistency and transparency in reporting. For further information on Nature Portfolio policies, see our Editorial Policies and the Editorial Policy Checklist.

Statistics

For all statistical analyses, confirm that the following items are present in the figure legend, table legend, main text, or Methods section.

n/a	Confirmed	
☒	☐	The exact sample size (*n*) for each experimental group/condition, given as a discrete number and unit of measurement
☒	☐	A statement on whether measurements were taken from distinct samples or whether the same sample was measured repeatedly
☒	☐	The statistical test(s) used AND whether they are one- or two-sided<br>*Only common tests should be described solely by name; describe more complex techniques in the Methods section.*
☒	☐	A description of all covariates tested
☒	☐	A description of any assumptions or corrections, such as tests of normality and adjustment for multiple comparisons
☐	☒	A full description of the statistical parameters including central tendency (e.g. means) or other basic estimates (e.g. regression coefficient) AND variation (e.g. standard deviation) or associated estimates of uncertainty (e.g. confidence intervals)
☒	☐	For null hypothesis testing, the test statistic (e.g. *F*, *t*, *r*) with confidence intervals, effect sizes, degrees of freedom and *P* value noted<br>*Give P values as exact values whenever suitable.*
☒	☐	For Bayesian analysis, information on the choice of priors and Markov chain Monte Carlo settings
☒	☐	For hierarchical and complex designs, identification of the appropriate level for tests and full reporting of outcomes
☒	☐	Estimates of effect sizes (e.g. Cohen's *d*, Pearson's *r*), indicating how they were calculated

Our web collection on statistics for biologists contains articles on many of the points above.

Software and code

Policy information about availability of computer code

Data collection	No software was used.
Data analysis	Microsoft Excel, QGIS 3.16.3, and Python 3.7.3 were used to analyze data.

For manuscripts utilizing custom algorithms or software that are central to the research but not yet described in published literature, software must be made available to editors and reviewers. We strongly encourage code deposition in a community repository (e.g. GitHub). See the Nature Portfolio guidelines for submitting code & software for further information.

Data

Policy information about availability of data

All manuscripts must include a data availability statement. This statement should provide the following information, where applicable:

- Accession codes, unique identifiers, or web links for publicly available datasets
- A description of any restrictions on data availability
- For clinical datasets or third party data, please ensure that the statement adheres to our policy

All data generated in this study have been provided in the Supplementary Materials and deposited in GitHub [https://github.com/PEESEgroup/Virtual_Con] with the published version archived (https://doi.org/10.5281/zenodo.5515049). Microsoft Excel (version 2109), QGIS 3.16.3, and Python 3.7.3 were used to analyze data. The LCIA data used in this study are available in the Ecoinvent database [https://ecoinvent.org/the-ecoinvent-database/] and the food supply data used in this study are available in the FAOSTAT database [https://www.fao.org/faostat/en/#data].

Field-specific reporting

Please select the one below that is the best fit for your research. If you are not sure, read the appropriate sections before making your selection.

☐ Life sciences ☐ Behavioural & social sciences ☒ Ecological, evolutionary & environmental sciences

For a reference copy of the document with all sections, see nature.com/documents/nr-reporting-summary-flat.pdf

Ecological, evolutionary & environmental sciences study design

All studies must disclose on these points even when the disclosure is negative.

Study description	A life cycle assessment study of virtual, in-person, and hybrid conferences.
Research sample	The participant's geographical information are obtained from 2020 ACLCA virtual conference.
Sampling strategy	All participants with geographical information are included in the analysis.
Data collection	Geographic information of participants was collected from the 2020 ACLCA conference by Debbie Steckel. Characterization factors was collected from the Ecoinvent database by Yanqiu Tao. Life cycle inventory data was collected by Yanqiu Tao from various sources as suggested in the manuscript.
Timing and spatial scale	Data is for 2020 and spatial scale is provided in Supplementary Table S2.
Data exclusions	No data were excluded from the analyses.
Reproducibility	All data needed to evaluate the conclusions in the paper are present in the paper or can be accessed through the Supplementary Materials and GitHub (https://github.com/PEESEgroup/Virtual_Con) with the published version archived (https://doi.org/10.5281/zenodo.5515049).
Randomization	No randomization was necessary because we include all ACLCA participants with geographical information in the analysis.
Blinding	Blinding is not relevant to this study because the results used for calculating the life cycle environmental impacts are computed from an optimization model and no subjective outcomes are involved.

Did the study involve field work? ☐ Yes ☒ No

Reporting for specific materials, systems and methods

We require information from authors about some types of materials, experimental systems and methods used in many studies. Here, indicate whether each material, system or method listed is relevant to your study. If you are not sure if a list item applies to your research, read the appropriate section before selecting a response.

Materials & experimental systems

n/a	Involved in the study	
☒	☐	Antibodies
☒	☐	Eukaryotic cell lines
☒	☐	Palaeontology and archaeology
☒	☐	Animals and other organisms
☒	☐	Human research participants
☒	☐	Clinical data
☒	☐	Dual use research of concern

Methods

n/a	Involved in the study	
☒	☐	ChIP-seq
☒	☐	Flow cytometry
☒	☐	MRI-based neuroimaging
